# Prevalence and determinants of anemia among people living with HIV receiving antiretroviral therapy in public health facilities in Jigjiga, Ethiopia

**DOI:** 10.1186/s12981-026-00912-2

**Published:** 2026-06-26

**Authors:** Jamaal Ibrahim, Sahardiid Abdilahi, Mohamed Osman

**Affiliations:** 1 Department of Clinical Nursing, Jigjiga Referral Comprehensive Hospital, Jigjiga University, P.O. Box 1020, Jigjiga, Ethiopia; 2 School of Public Health, Institute of Health Sciences, Jigjiga University, P.O. Box 1020, Jigjiga, Ethiopia; 3 Graduate School of Medicine, University of Wollongong, Wollongong, NSW 2522 Australia

**Keywords:** Anemia, HIV, CD4 count, Antiretroviral therapy, Jigjiga, Ethiopia

## Abstract

**Background:**

Anemia is a significant concern for people with HIV worldwide, as it is associated with reduced life expectancy, fatigue, weakness, reduced physical functioning, psychological distress such as depression and anxiety, and poorer quality of life. This study aimed to assess the prevalence of anemia and identify factors associated with anemia among people with HIV receiving antiretroviral therapy at public health institutions in Jigjiga, Ethiopia.

**Methods and materials:**

An institution-based cross-sectional study was conducted from July 11 to August 12, 2023, involving 392 people with HIV receiving antiretroviral therapy in Jigjiga, Ethiopia. Data were collected using a structured questionnaire and hemoglobin measurements, then coded and entered in EpiData version 3.1. Statistical analysis was performed using SPSS version 20, with multivariate logistic regression used to identify factors associated with anemia. Statistical significance was set at *p* < 0.05.

**Results:**

The overall prevalence of anemia in the study population was 39% (95% CI: 34.3–44.2). Among those with anemia, 0.6% had severe anemia, 33.5% had moderate anemia, and 4.9% had mild anemia. Female sex, presence of opportunistic infections, and low dietary diversity scores were significantly linked to anemia in multivariate analysis (*p* < 0.05).

**Conclusion:**

Anemia was identified as a moderate public health problem among people with HIV receiving ART at the study sites. The findings highlight the importance of providing targeted support to women and ensuring timely diagnosis and treatment of opportunistic infections to reduce the severity and impact of anemia. Furthermore, patients with poor dietary diversity should be offered nutritional counselling to enhance their health outcomes.

**Significance for public health:**

Anemia continues to pose a substantial public health burden among people living with HIV/AIDS, particularly in low-resource settings. This study provides context-specific evidence from Jigjiga, a region with limited existing data, helping to fill a critical knowledge gap. The results offer valuable insights for healthcare providers and policymakers to design targeted interventions, improve anemia screening and management, and enhance the quality of life for People with HIV on ART.

## Introduction

### Background

Globally, HIV infection remains a significant public health concern and substantially contributes to morbidity and mortality. Since the beginning of the epidemic, an estimated 76.1 million individuals have been infected with the virus, and 35.0 million have died from illnesses linked to acquired immunodeficiency syndrome (AIDS) [1]. A total of 36.7 million People with HIV, 1.8 million new HIV infections and one million AIDS related deaths occurred worldwide in 2016. In 2015, Sub-Saharan Africa (SSA) accounted for 76% of People Living with HIV, 76% of new HIV infections, and 75% of HIV related deaths worldwide [2].

Ethiopia continues to have a significant HIV burden, despite significant improvements in HIV interventions during the Millennium Development Goals (MDG) period. According to the 2017 UNAIDS Global AIDS Report, an estimated 718,500 Ethiopians had HIV in 2016, compared to approximately 1.2 million in 2010, suggesting a decrease over time [3, 4].

Anaemia is a common haematological disorder in people living with HIV/AIDS. Anaemia leads to reduced physical performance in People Living with HIV, reduced quality of life, shorter life expectancy, psychological distress such as anxiety and depression, and accelerated HIV disease progression. Further, it is a serious health problem worldwide, which can have a negative effect on the socio-economic development and the standard of living of a country, leading to mortality and morbidity. Developing countries are more affected than developed countries because they often do not have the healthcare infrastructure and resources needed to manage it effectively, and developing regions have less access to early diagnosis, effective treatment and supportive care, leading to higher rates of complications and mortality from both conditions [5–8].

The prevalence of anaemia among People Living with HIV receiving antiretroviral therapy varies across different studies, ranging from 24% to 58% in Africa and between 23% and 50% worldwide; systematic reviews and meta-analyses report a pooled prevalence of 46.6% among adults living with HIV [9]. Anaemia has been reported to be more frequent in newly initiated ART patients, but the findings are not consistent across studies. Anaemia was reported in up to 35% of People Living with HIV who had not initiated ART in Ethiopia. However, one study in southwest Ethiopia found an anaemia prevalence of 16.2% among newly initiated ART patients and 29.9% among ART experienced patients [10]. This difference between studies may be related to differences in baseline clinical status, duration of ART, nutritional status, opportunistic infections and ART regimen, particularly zidovudine use, which is no longer Ethiopia’s first-line ART but remains relevant in some clinical settings [11, 12].

The main causes of anemia include inadequate nutrition, infections, and genetic abnormalities; among women who are of reproductive age, menstrual blood loss and postpartum haemorrhage are also significant contributing factors [13]. The cause of anemia in adult with HIV patients may vary depending on several factors. A few of these concerns are iron deficiency anemia (IDA), hookworm infection, sickle cell disorders, thalassemia, schistosomiasis, malaria, duration of antiretroviral therapy (ART), history of anti-tuberculosis (TB) drug treatment, presence of opportunistic infections (OIs), advanced stage of HIV disease, and socioeconomic and demographic factors such as marital status, educational level, income level, sex, age and residence may be associated with anaemia through their impact on nutrition, access to health care and living conditions. In addition, a low white blood cell count (< 4,000/µL), a low CD4 + T lymphocyte count (< 200/µL), and a low platelet count (< 200,000/µL) are the recommended cut off values for each or haematological abnormality [14, 15].

Treating anemia has been shown to improve survival rates and enhance the quality of life for people with HIV. Management of anemia is essential and should be tailored based on its underlying cause and type. Proper identification and treatment of anemia among people with HIV remains a critical public health priority [16].

Although several studies have been conducted on anemia among people living with HIV in other parts of Ethiopia including Amhara, Oromia, Tigray and Southern regions of Ethiopia, evidence on Somali Regional State of Ethiopia is limited [17]. Specifically, no previous study has been conducted the prevalence and determinants of anaemia among people living with HIV in Jigjiga, a predominantly pastoralist setting. Given the continuing dual burden of HIV and anemia in Ethiopia, context-specific evidence is needed to inform local HIV care and anaemia management strategies.

Therefore, this study aimed to assess the prevalence of anemia and determinant factors among people living with HIV receiving antiretroviral therapy at public health institutions in Jigjiga, Ethiopia.

## Methods

### Study design and period

An institution-based cross-sectional study was conducted in Jigjiga from July 11 to August 12, 2023. Jigjiga is located 626 km east of Addis Ababa, the capital city of Ethiopia. As of the 2023 Ethiopian fiscal year, Jigjiga has a total population of 426,122 individuals, 85,650 of whom are in the reproductive age group (15–49 years). The majority of the population are ethnic Somalis (97%) and Muslims (98%) [18]. However, the present study was facility-based and included ART clients attending selected health facilities. The city is served by a range of healthcare facilities, including one comprehensive specialised teaching hospital, one general hospital, one primary hospital, three health centres, one private hospital, and eight clinics providing ART services.

### Source and study population

#### Source population

All adult patients aged 18 years and above living with HIV who were residing in Jigjiga, Ethiopia.

#### Study population

All randomly selected adult patients with HIV who were attending the selected public health institutions during data collection at Jigjiga, Ethiopia, were included. Adult patients with HIV who were pregnant or severely ill were excluded from the study.

#### Sample size determination and sampling procedure

The sample size was determined using the single population proportion formula in OpenEpi version 7, considering an estimated anemia prevalence of 36.5% [19], a 95% confidence level, a 5% margin of error. This resulted in a required sample size of 356 participants. To account for a potential 10% nonresponse rate, the final sample size was increased to 392.

To ensure representative sampling across all public health facilities providing ART services in Jigjiga, a multistage stratified sampling method was employed. The total sample was allocated to each facility proportionally based on the number of people living with HIV enrolled in ART care. Within each facility, a list of eligible patients scheduled for routine follow-up during the data collection period was compiled. From these lists, participants were randomly selected using simple random sampling. On their scheduled appointment day, trained data collectors approached the selected patients in the clinic waiting areas, explained the study objectives, and obtained written informed consent. In cases where a selected individual declined participation or was found ineligible, the next randomly selected patient was approached to maintain the required sample size. A total of 392 participants consented and were enrolled in the study.

### Data collection instrument and procedure

#### Data collection tools

A standardized, pretested questionnaire in Somali was used to collect data, focusing on clinical and sociodemographic factors, as well as a 24-hour dietary recall developed after a thorough review of the literature. Following standard operating procedures, the ring finger was cleaned with 70% ethanol, allowed to air dry, and punctured with a sterile lancet to obtain a capillary blood sample for measuring hemoglobin concentration and determining anemia status. Data were collected by three medical laboratory technicians working in public health institutions and supervised by one bachelor-level health officer.

#### Measurements

Anemia was defined using the World Health Organization (WHO) criteria, with hemoglobin thresholds adjusted for sex, altitude, and smoking status. Individuals with haemoglobin levels below 13.0 g/dL (for men) and below 12.0 g/dL (for non-pregnant women) were classified as anaemic, while its severity was categorized as mild anemia (11.0–12.9 g/dL in men; 11.0–11.9 g/dL in non-pregnant women), moderate anemia (8.0–10.9 g/dL), and severe anemia (less than 8.0 g/dL) according to WHO hemoglobin cutoffs [20].

Residence was self-reported and categorised according to the administrative classification of the Central Statistical Agency of Ethiopia. Urban areas were considered those localities that were formally recognised as towns or cities with municipal administration. Rural areas were all the areas outside the urban areas [21].

Dietary diversity was measured using a 24-hour recall questionnaire adapted from the Food and Agriculture Organization (FAO) guidelines [22]. The recall covered nine food groups commonly consumed in the local context: (1) cereals, roots, and tubers; (2) legumes and nuts; (3) dairy products; (4) meat, poultry, and fish; (5) eggs; (6) dark green leafy vegetables; (7) other vitamin A-rich fruits and vegetables; (8) other fruits; and (9) oils and fats.

Participants received one point for each food group consumed in the previous 24 h, resulting in a total dietary diversity score (DDS) ranging from 0 to 9. Based on FAO recommendations and evidence from prior studies in similar settings, dietary diversity was categorized as follows:

Good dietary diversity: ≥5 food groups and Poor dietary diversity: <5 food groups.

Intestinal parasitic infection was defined as the presence of one or more intestinal parasites detected in stool samples by direct wet mount and formol-ether concentration techniques. The parasites included Ascaris lumbricoides, Trichuris trichiura, hookworm species, Entamoeba histolytica/dispar and Giardia lamblia.

Opportunistic illnesses were defined as clinically diagnosed conditions documented in the patients’ medical records. These included tuberculosis, oral candidiasis, chronic diarrhoea, bacterial pneumonia, herpes zoster and other AIDS defining illnesses.

#### Data quality management

To ensure the quality of the data, both primary and secondary patient data were included in the process. Two days of training focusing on the objectives of the study, measurement procedures, ethical issues, and standardization of intra- and interobserver variation were provided to the data collectors. Data collectors, supervisors, and the principal investigator had daily discussions following data collection to discuss issues that arose and how they were resolved through a shared memorandum of understanding. A pretest and demonstration of the instrument were performed on 5% of the sample in similar communities that were not included in the sample. To ensure accuracy and consistency, questionnaires were prepared in English, translated into Somali and Amharic, and then returned into English.

### Study variables

#### Dependent variable

Anemia status is based on the hemoglobin level.

#### Independent variables

Sociodemographic characteristics (sex, occupation, education level, monthly income), and clinical characteristics (BMI, ART regimen, TB treatment, CD4 count, intestinal parasites, HAART duration, WHO clinical stage, dietary diversity score, malaria parasites, and cotrimoxazole use) were recorded.

#### Data analysis procedures

The data were entered, cleaned, and coded using EpiData version 3.1 to address inconsistencies and missing values, and subsequently analyzed using SPSS version 24. Descriptive statistics, including frequencies, proportions, means, and standard deviations, were computed. Bivariate and multivariate logistic regression analyses were performed to identify independent predictors of anemia, with odds ratios (ORs) and 95% confidence intervals (CIs) reported. Variables with a p-value ≤ 0.25 in the bivariate analysis were included in the multivariate logistic regression model. A p-value of < 0.05 was considered statistically significant. Model adequacy and fitness were assessed using the Hosmer–Lemeshow goodness-of-fit test, and all assumptions of logistic regression, including model adequacy and multicollinearity among independent variables, were examined.

## Results

### Sociodemographic characteristics of the participants

A total of 392 People Living with HIV and receiving antiretroviral therapy were recruited for the study. Of these, 385 consented to participate and completed the interview, resulting in a response rate of 98.2%. Among the participants, 65.5% were women, and the mean age of participants was 40 years. Most (67.8%) were between 31 and 49 years old, highlighting that HIV predominantly affects people in their prime working years. Nearly half (46.0%) were married, with widowed and divorced participants making up a significant portion. The majority identified as Muslim (44.9%), followed by Orthodox Christians (29.1%) and Protestants (19.5%). Education levels were low, with nearly half having no formal education or only basic literacy. 24.4% were daily laborers, followed by merchants (22.1%). Most participants (95.6%) lived in urban areas (Table 1).

**Table 1:** Sociodemographic characteristics of the participants

Varaibles	Category	Frequency	Percentage (%)	Anemia(150)	Non anemia (235)
Age	18–30	87	22.6	32 (36.8)	55 (63.2)
	31–49	261	67.8	105 (40.2)	156 (59.7)
	> 50	37	9.6	13 (35.1)	24 (64.9)
Sex	Male	133	34.5	31 (23.3)	102 (76.7)
	Female	252	65.5	119 (47.2)	133 (52.7)
Marital status					
	Single	83	21.6	31	52
	Married	177	46	54	123
	Divorced	85	22	40	45
	Widowed	40	10.4	25	15
Religion	Muslim	173	44.9	72 (41.6)	101 (58.4)
	Orthodox	112	29.1	47 (42)	65 (58)
	Protestant	75	19.5	31 (41.3)	44 (58.7)
	Catholic	24	6.2	9 (37.5)	15 (62.5)
	Other	1	0.3	0	0
Education level	No formal Education	83	21.6	34 (40.9)	49 (59.1)
	Read and write	95	24.7	39 (41)	56 (59)
	Primary school	74	19.2	30 (40.5)	44 (59.5)
	High school	72	18.7	27 (37.5)	45 (62.5)
	Diploma and above	61	15.8	20 (33)	41 (67)
Occupation	Student	28	7.3	11 (39.3)	17 (60.7)
	Private business	69	17.9	25 (36.2)	44 (63.8)
	House wife	73	19	29 (39.7)	44 (60.3)
	Daily laborer	94	24.4	39 (41.5)	55 (58.5)
	Merchant	85	22.1	31 (36.5)	54 (63.5)
	Other	36	9.3	15 (41.6)	21 (58.3)
Residence	Urban	367	95.6	138 (37.6)	229 (62.4)
	Rural	18	4.4	12 (66.6)	6 (33.3)

### Clinical characteristics of the participants

Among 385 People Living with HIV on antiretroviral therapy, 73.3% had experienced opportunistic infections, and 15.1% tested positive for intestinal parasites. Most participants (80.3%) had a normal body mass index (18.5 to 24.9 kg/m²), while 10.4% were underweight (less than 18.5 kg/m²) [23]. Nearly all (95.8%) were on non-zidovudine regimens, primarily the national first-line therapy of tenofovir, lamivudine, and efavirenz. Tuberculosis treatment was reported in 13.2% of participants. Almost half (48.1%) had been on HAART therapy for 6 to 30 months. CD4 counts revealed that 8.8% had fewer than 200 cells/mm³, and 37.1% had counts between 201 and 350 cells/mm³. Malaria was uncommon (2.3%), but poor dietary diversity was noted in 67%, indicating potential nutritional concerns (Table 2).

**Table 2:** Clinical characteristics of study participants living with HIV

Variables	Category	Frequency	Percentage (%)	Anemia (150)	Non anemia (235)
Opportunistic infection	Yes	282	73.3	129 (45.7)	153 (54.3)
	No	103	26.7	21 (20.4)	82 (79.6)
Intestinal parasite	Positive	58	15.1	25 (43.1)	33 (56.9)
	Negative	327	84.9	125 (38.2)	202 (61.8)
Body mass index	Underweight(< 18.5)	40	10.4	16 (40.0)	24 (60.0)
	Normal(18.5–24.9)	309	80.3	121(39.1)	188 (60.9)
	Overweight(25.0–29.9)	36	9.3	13 (36.1)	23 (63.9)
ART regimens	ZDV containing	16	4.2	7 (43.7)	9 (56.3)
	No ZDV containing	369	95.8	143 (38.8)	226 (61.2)
Anti Tb treatment	Yes	51	13.2	21 (41.2)	30 (58.8)
	No	334	86.8	129 (38.6)	205 (61.4)
Duration of HAART	6–30 months	185	48.1	64 (34.6)	121 (65.4)
	31–60 months	139	36.1	58 (41.7)	81 (58.3)
	61–90 months	25	6.5	9 (36.0)	16 (64.0)
	> 90 months	36	9.3	19 (52.8)	17 (47.2)
CD4 count (cells/µL)	< 200	34	8.8	15 (41.6)	19 (55.9)
	201–350	143	37.1	56 (39.2)	87 (60.8)
	351–500	122	31.7	46 (37.7)	76 (62.3)
	> 500	86	22.3	33 (38.4)	53 (61.6)
Malaria	Positive	9	2.3	3 (33.3)	6 (66.7)
	Negative	376	97.7	147 (39.1)	229 (60.9)
Dietary diversity score	Good	127	33	30 (23.6)	97 (76.3)
	Poor	258	67	120 (46.5)	138 (53.5)

Includes intestinal parasites detected by stool examination using direct wet mount and/or concentration techniques

### Prevalence and severity of anemia

**Fig. 1:**
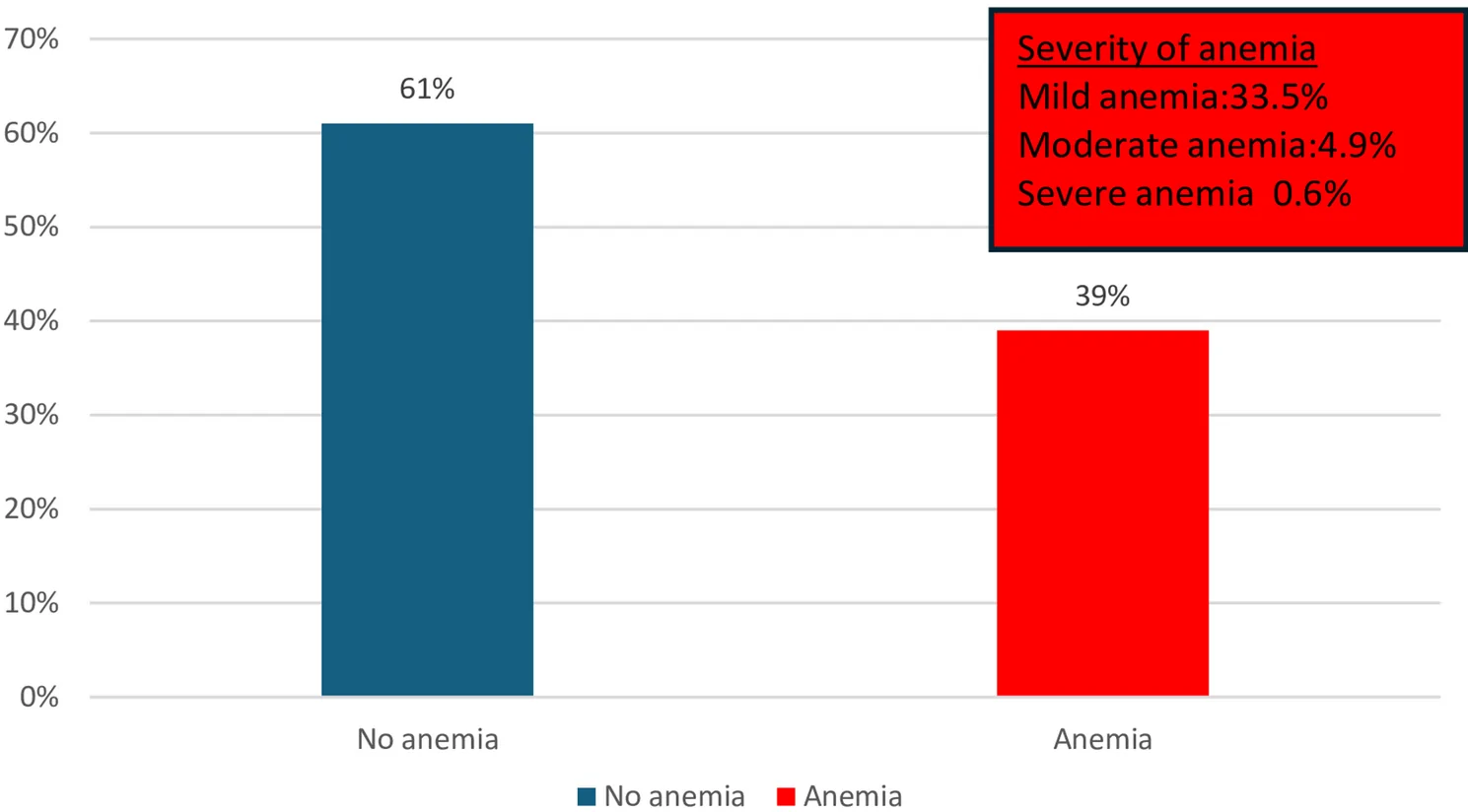
Prevalence and severity of anemia among study participants

Among the study participants, the prevalence of anemia was 39% (95% CI: 34.3–44.2%). Mild anemia was the most common, affecting 33.5% of participants, followed by moderate anemia (4.9%). Severe anemia (0.6%) was rare (Fig. 1).

### Factors associated with anemia

Multivariate logistic regression analysis showed that female participants were significantly more likely to experience anemia compared to males (AOR = 2.1; 95% CI: 1.2–3.5, *p* ≤ 0.05). Participants living in urban areas had higher odds of anemia than those in rural settings (AOR = 2.3; 95% CI: 1.8–6.9, *p* ≤ 0.05). The presence of opportunistic infections was strongly associated with anemia, with affected individuals having more than twice the odds compared to those without infections (AOR = 2.3; 95% CI: 1.3–4.1, *p* ≤ 0.05). Additionally, poor dietary diversity significantly increased the likelihood of anemia, doubling the odds relative to participants with adequate dietary diversity (AOR = 2.4; 95% CI: 1.4–4.0, *p* ≤ 0.05) (Table 3).

**Table 3:** Factors associated with anemia among study participants

Variables	Anemia		COR (95%CI)	AOR (95%CI)
	Yes (%)	No (%)		
Sex				
Male	31	102	1	1
Female	119	133	2.9 (1.8–4.7)*	2.1 (1.2–3.5)**
Marital status				
Single	31	52	1	1
Married	54	123	0.73 (0.42–1.27)	1.2 (0.8–5.6)
Divorced	40	45	1.4 (0.8–2.7)*	1.3 (0.6–2.5)
Widowed	25	15	2.7 (1.3–6.1)*	1.1 (0.9–5.9)
Residence				
Urban	138	229	3.3(1.2-9.0)*	2.3 (1.8–6.9) **
Rural	12	6	1	1
Opportunistic infection				
Yes	129	153	3.2 (1.8–5.5)*	2.3 (1.3–4.1)**
No	21	82	1	1
Duration of HAART (months)				
6–30	64	121	1	1
31–60	58	81	1.9 (1.86–2.1)*	1.2 (0.7-2.0)
61–90	9	16	1.0 (0.44–2.5)	1.1 (0.21–3.8)
> 90	19	17	2.1 (1.0-4.3)	1.3 (0.38–6.7)
Dietary diversity score				
Good	30	97	1	1
Poor	120	138	2.8 (1.7–4.5) *	2.4 (1.4-4.0) **

NB: 1 refference * Significant at value < 0.25, ** Significant at p-value of ≤ 0.05, COR: crude odds ratio, AOR: adjusted odds ratio

## Discussion

This study found that the prevalence of anemia among People Living with HIV receiving ART was 39% (95% CI: 34.3–44.2), indicating a moderate public health problem in the study setting, according to WHO threshold criteria (20–39%) [24]. Anemia was significantly associated with female sex, urban residence, the presence of opportunistic infections, and poor dietary diversity.

The findings of this study on the prevalence of anemia among People Living with HIV receiving ART were consistent with similar research on people living with HIV conducted in the USA, which reported a prevalence of 39.6% [25]; Sawla General Hospital, Southern Ethiopia (38.6%) [26]; Wolaita Sodo University (36.5%) [27]^;^ and Gondar University Hospital, Northwest Ethiopia (35%) [28]. These findings are higher than those of studies conducted at Debre Berhan Referral Hospital, Ethiopia (26.2%) [29], South Africa (25.8%) [30], Debre Tabor Hospital, Ethiopia (23%) [31]. However, they are lower than those reported in studies conducted in Arba Minch Town, Southern Ethiopia (52.3%) [32], Uganda (47.8%) [33] and Indonesia (49.6%) [34]. Differences in sociodemographic characteristics, geographic location, time of study, healthcare access, and the implementation of HIV related interventions may account for these variations in prevalence. In addition, differences in anemia definitions, adjustments for altitude and smoking, and variations in ART protocols across settings may also contribute to the differences observed.

The region is predominantly pastoralist and a drought-prone region, where food insecurity, mobility and limited access to health services may affect anemia and HIV outcomes differently than in other regions in Ethiopia [35]. In this context, the continuity of treatment and nutritional vulnerability may be affected by socio cultural practices, cross border population movement, and interruptions in access to routine HIV services.

According to this study, females were 2.1 times more likely than males to develop anemia, which aligns with findings from other studies conducted both within and outside the country, including research from USA [36] and South Africa [30], Debre Tabor Hospital, Northwest Ethiopia [37], and USA [38]. This increased risk is most likely due to the greater blood loss experienced by pregnant women and women of reproductive age during menstrual cycles, making them more susceptible to iron-deficiency anemia. Additionally, pregnant women who do not take multivitamins containing folic acid and iron are at higher risk of anemia, as pregnancy significantly increases iron requirements to support both maternal health and fetal development. These physiological demands likely explain the observed sex disparity in anemia prevalence. Men typically store approximately 1000 mg of iron, whereas women store between 300 and 500 mg. Additionally, women lose between 12.5 and 15.0 mg of iron monthly through menstrual blood [37]. However, some studies reported different results, with some found no significant difference in anemia prevalence between females and males [17, 39], while some others suggested that men are more likely to develop anemia than females [30, 32]. These inconsistent findings could partly be explained by age distribution as anemia increases with age in men but is more common during the reproductive years and may decline after menopause in women [40, 41]. However, further research is needed to explore the association between anemia and sex among People with HIV/AIDS.

In this study, urban residence was significantly associated with anemia among people with HIV, and urban residents were 2.3 times more likely to have anemia compared to those who reside in rural areas. This study was supported by a study done in Malawi, which reported the prevalence of anemia was higher among urban residents [42]. This may be because iron deficiency may be less prevalent in rural areas, as rural residents have better access to diverse and more nutritious food sources: foods rich in iron, such as eggs, leafy green vegetables, and, in our lakeshore population, fish; and fruits containing vitamin C, which enhances iron absorption. Rural women may have improved access to the mineral-rich clay pellets traditionally consumed during pregnancy [43].

The present study revealed that individuals with opportunistic infections were 2.3 times more likely to develop anemia compared to those without such opportunistic infections. This finding is consistent with previous studies conducted in the Kembata Tembaro Zone of southern Ethiopia [44], Supported by a meta-analysis and comprehensive review [9], a narrative literature review [45], and a systematic review on the HIV situation in Addis Ababa, Ethiopia [46]. The observed association may be explained by the inflammatory response triggered by opportunistic infections, which stimulates the release of pro-inflammatory cytokines. These cytokines can impair iron metabolism by reducing its absorption and sequestration, interfering with erythropoietin production, and ultimately disrupting red blood cell formation, all of which contribute to the development of anemia. In the present study, CD4 count was not significantly associated with anemia, however opportunistic infections have shown more significant association.

In this study, opportunistic infections were analysed as a composite variable (yes/no) and were significantly associated with anemia (*P* < 0.05). However, because of the small sample size, we did not assess specific types of opportunistic infections separately in the current study. Thus, it was not possible to determine if specific disease like tuberculosis, oral candidiasis or chronic diarrhoea were more significantly associated with an anemia. The most reported opportunistic infections among the participants in this study were pneumonia, chronic diarrhoea, oral candidiasis, and tuberculosis. This pattern is in line with findings of previous studies done in Ethiopia [47].

This study found that individuals with poor dietary diversity were 2.4 times more likely to develop anemia compared to those with good dietary diversity. This finding is consistent with research conducted at Debre Tabor Hospital in Northwest Ethiopia [37]. This might be that diversified diets rich in micronutrients such as iron, folate, and vitamin B12 are required for hemoglobin production and may help avoid anemia, particularly among those living with HIV who may already face heightened nutritional vulnerabilities. Our findings contrast with research carried out in Wolaita Sodo, Ethiopia that found no significant association between anemia and dietary diversity [48]. The difference may be explained by differences in the research setting, food availability, socioeconomic conditions, dietary habits, and the proportion of people with adequate nutritional support or urban access to a range of foods.

### Limitations

This study has several limitations. First, because of the cross-sectional design, causal associations between anemia and its associated factors cannot be established. Second, data collection coincided with the COVID-19 pandemic, during which many patients received multi-month ART prescriptions to reduce clinic visits, potentially affecting the representativeness of the sample. Third, results are generalisable only to people living with HIV who are receiving HIV care and are taking ART and exclude those who are newly infected and did not attend health care facilities and those who are not engaged in care. Fourth, data were gathered during the Orthodox Christian fasting period, which may have influenced participants’ dietary patterns and subsequently affected the assessment of dietary diversity. Fifth, social desirability bias and fear of stigma may have led some participants to underreport or misreport sensitive information. Sixth, social desirability bias, recall bias, and other self-reported data may have introduced reporting bias.

Finally, the absence of an HIV negative comparison group limits our ability to determine whether the observed associations are specific to people living with HIV or generalizable to the wider population.

## Conclusion

This study found anemia as a moderate public health problem among People Living with HIV in the study area, according to the WHO classification of anemia as a public health problem (20–39%) and the reported prevalence was 39%. Anemia was significantly associated with female sex, the presence of opportunistic infections, and poor dietary diversity factors that can negatively impact treatment outcomes and increase the risk of morbidity and mortality.

This study suggested strengthening targeted anemia screening, nutritional counselling, dietary assessments and iron supplementation in HIV services especially in Jigjiga city, Ethiopia. Furthermore, early detection and early management of opportunistic infections should be included in routine HIV care.

Longitudinal cohort studies are recommended to further explore the long term relationship between HIV related factors and anemia, and to inform the development of sustainable, evidence-based interventions.

## Data Availability

No datasets were generated or analysed during the current study.

## Abbreviations

AIDS: Acquired immune deficiency syndrome
AOR: Adjusted odds ratio
ART: Antiretroviral therapy
BMI: Body mass index
CD4: Cluster of differentiation 4
COR: Crude odds ratio
HAART: Highly active antiretroviral therapy
HIV: Human immunodeficiency virus
OI: Opportunistic infection
SD: Standard deviation
SPSS: Statistical package for the social sciences
TB: Tuberculosis
WHO: World health organization
ZDV: Zidovudine
